# Current Therapeutic Landscape for Metabolic Dysfunction-Associated Steatohepatitis

**DOI:** 10.3390/ijms26041778

**Published:** 2025-02-19

**Authors:** Arun George Devasia, Adaikalavan Ramasamy, Chen Huei Leo

**Affiliations:** 1 Science, Math & Technology, Singapore University of Technology & Design, Singapore 487372, Singapore; devasiaag@gis.a-star.edu.sg; 2 Genome Institute of Singapore (GIS), Agency for Science Technology and Research (A*STAR), 60 Biopolis Street, Singapore 138672, Singapore; adai@gis.a-star.edu.sg; 3 Department of Biomedical Engineering, College of Design & Engineering, National University of Singapore, 9 Engineering Drive 1, Singapore 117576, Singapore

**Keywords:** metabolic dysfunction-associated steatotic liver disease, metabolic dysfunction-associated steatohepatitis, therapeutics, non-alcoholic fatty liver disease

## Abstract

In recent years, “metabolic dysfunction-associated steatotic liver disease” (MASLD) has been proposed to better connect liver disease to metabolic dysfunction, which is the most common chronic liver disease worldwide. MASLD affects more than 30% of individuals globally, and it is diagnosed by the combination of hepatic steatosis and obesity, type 2 diabetes, or two metabolic risk factors. MASLD begins with the buildup of extra fat, often greater than 5%, within the liver, causing liver hepatocytes to become stressed. This can proceed to a more severe form, metabolic dysfunction-associated steatohepatitis (MASH), in 20–30% of people, where inflammation in the liver causes tissue fibrosis, which limits blood flow over time. As fibrosis worsens, MASH may lead to cirrhosis, liver failure, or even liver cancer. While the pathophysiology of MASLD is not fully known, the current “multiple-hits” concept proposes that dietary and lifestyle factors, metabolic factors, and genetic or epigenetic factors contribute to elevated oxidative stress and inflammation, causing liver fibrosis. This review article provides an overview of the pathogenesis of MASLD and evaluates existing therapies as well as pharmacological drugs that are currently being studied in clinical trials for MASLD or MASH.

## 1. Introduction

**Figure 1:**
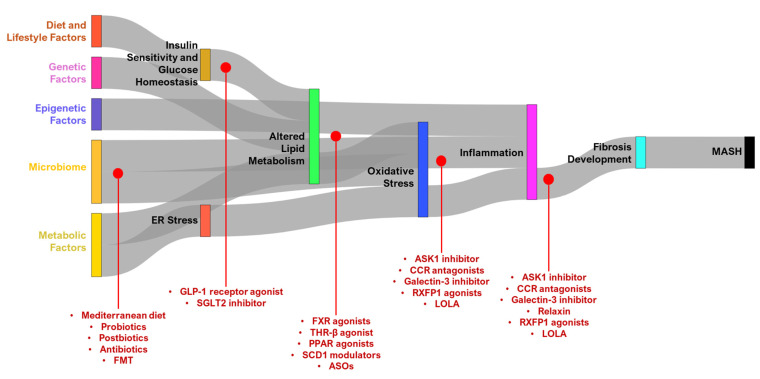
An overview of the pathogenesis and therapeutics of MASH. Multiple factors synergistically interact in a cascade of events, ultimately leading to liver damage and disease progression. FMT = fecal microbiota transplantation; GLP-1 = glucagon-like peptide-1; SGLT2 = sodium–glucose cotransporter 2; FXR = farnesoid X receptor; THR-β = thyroid hormone receptor beta; PPAR = peroxisome proliferator-activated receptor; SCD1 = stearoyl-CoA desaturase 1; ASOs = antisense oligonucleotides; ASK1 = apoptosis signal-regulating kinase 1; CCR = C-C chemokine receptor; RXFP1 = relaxin/insulin-like family peptide receptor 1; LOLA = L-ornithine L-aspartate.

Metabolic dysfunction-associated steatotic liver disease (MASLD), previously known under the broader category of non-alcoholic fatty liver disease (NAFLD), represents a significant shift in how liver diseases related to metabolic dysfunction are understood, diagnosed, and treated [1]. MASLD, which includes the more severe form known as metabolic dysfunction-associated steatohepatitis (MASH), is now recognized as a condition inherently linked to metabolic risk factors such as obesity, type 2 diabetes mellitus (T2DM), dyslipidemia, and insulin resistance [1]. However, MASLD emphasizes the presence of metabolic dysfunctions that drive this fat accumulation. The pathogenesis of MASLD involves complex interactions between genetic predisposition, environmental influences, and metabolic disturbances [2]. The “multiple-hit” hypothesis, which has evolved from the older “two-hit” model, describes how various factors such as insulin resistance, adipokine dysregulation, gut microbiota alterations, and lipotoxicity act synergistically to contribute to the progression of the disease [2], acknowledging that these processes are not sequential but rather simultaneous and interconnected [1]. This redefinition of MASLD is crucial because it more accurately reflects the underlying pathophysiology of the disease and broadens the diagnostic criteria to include metabolic risk factors such as type 2 diabetes, overweight/obesity, elevated waist circumference, high blood pressure, high triglycerides, low HDL cholesterol, prediabetes, insulin resistance, and increased levels of C-reactive protein [3]. Hence, this present review summarizes the current body of knowledge regarding the pathogenesis of MASH. Lastly, we also discuss the recent developments of effective therapeutic strategies that can address multiple pathogenic pathways simultaneously, including targeting lipid metabolism, reducing oxidative stress and inflammation, enhancing insulin sensitivity, and modulating the gut microbiome [4] (Figure 1).

## 2. Pathogenesis of MASH

The earlier “two-hit” hypothesis of MASH pathogenesis, proposed by James and Day in 1998, suggested that while obesity increases liver fat deposition, an additional “hit” was necessary to induce inflammation and fibrosis [5]. However, recent research has evolved towards the “multiple-hit” hypothesis, which provides a more comprehensive understanding of MASH pathogenesis [6]. This model posits that the disease arises from the synergistic effects of various concurrent factors, including metabolic disturbances, genetic predispositions, and environmental influences such as insulin resistance, adipokine dysregulation (such as reduced adiponectin and elevated pro-inflammatory cytokines like tumor necrosis factor alpha (TNF-α) and interleukin 6 (IL-6)), and gut microbiota alterations (dysbiosis), which all contribute to the development of MASLD and its progression to MASH [6]. These factors increase intestinal permeability, allowing bacterial endotoxins to enter the liver, thereby exacerbating inflammation [4]. Additionally, oxidative stress from an imbalance between reactive oxygen species (ROS) and antioxidant defenses damages cellular components, promoting further inflammation, cell death, and fibrosis [4]. Genetic variations, such as those in the PNPLA3 and TM6SF2 genes, along with epigenetic modifications, increase susceptibility to disease progression [7]. The dysregulation of cholesterol metabolism further exacerbates liver injury and fibrosis, while endothelial dysfunction in the liver’s sinusoidal blood vessels impairs hepatic blood flow, contributing to the advancement of fibrosis [8]. Endothelial dysfunction causes an imbalance in vascular homeostasis that switches to a vasoconstrictor state and lowers vasodilation [9,10,11]. Hence, the production and bioactivity of vasoprotective molecules such as nitric oxide, prostacyclin, and endothelium-derived hyperpolarization are impaired, due to vascular oxidative stress and inflammation [12,13,14,15]. Indeed, it has been reported that patients with MASH have concomitant liver fibrosis and systemic vascular dysfunction, contributing to increased risk for cardiovascular diseases [16,17,18]. This intricate pathophysiological process of MASLD not only drives liver disease but also heightens the cardiovascular risk associated with MASLD, making it a systemic condition that requires comprehensive management.

### 2.1. Diet and Lifestyle Factors of MASH

Diet plays a crucial role in the pathogenesis of MASH and MASLD, profoundly influencing metabolic health and body composition [19]. Excessive intake of calories, particularly from high-fat and high-sugar sources, results in caloric surplus, promoting weight gain and hepatic fat deposition [20]. Ultra-processed foods (UPFs) such as fried foods, processed snacks, baked goods, sugary beverages, and sweets, characterized by high energy density and elevated levels of saturated and trans fats, sugars, and additives, exacerbate these effects by directly increasing liver fat accumulation and stimulating liver-driven lipogenesis, especially in fructose-rich diets [21,22]. These nutrient-poor food products contribute to metabolic disturbances, including obesity, insulin resistance, inflammation, and visceral fat accumulation, key factors in the progression of MASH and MASLD [23]. Subjects with a high consumption of ultra-processed foods have low compliance with the Mediterranean diet [24]. Conversely, dietary patterns prioritizing the Mediterranean diet have demonstrated significant benefits in improving metabolic health and reducing central adiposity [24]. These findings underscore the critical need for dietary strategies that limit UPF intake while promoting nutrient-rich, minimally processed foods to prevent and manage these liver conditions effectively.

Choline is an essential nutrient pivotal in liver health, particularly in synthesizing phosphatidylcholine, a key component of cellular membranes and lipoproteins required for very low-density lipoprotein (VLDL) assembly and secretion [25]. Choline deficiency has been strongly implicated in the development of MASLD, as it disrupts VLDL secretion, leading to triglyceride accumulation in the liver, a hallmark of MASLD [26]. Mechanistically, inadequate choline intake reduces phosphatidylcholine synthesis, alters the phosphatidylcholine-to-phosphatidylethanolamine ratio, and impairs membrane integrity, activating lipogenesis via sterol regulatory element-binding proteins [27]. While choline deficiency alone cannot induce MASLD, it may act synergistically with other factors, such as methionine deficiency or gut dysbiosis, to promote inflammation and fibrosis.

Various lifestyle factors also play a crucial role in influencing the susceptibility and progression of MASH, closely intertwining with dietary patterns. Insufficient physical activity represents a notable risk factor as it contributes to obesity and insulin resistance, key players in the pathophysiology of MASH [28]; hence, regular exercise is imperative for reducing hepatic fat content and enhancing insulin sensitivity [29]. Although MASH can develop in non-alcoholic individuals, moderate alcohol consumption can exacerbate liver damage in those predisposed to or already afflicted by MASH, as well as contribute to metabolic dysfunction and alcohol-related liver disease (MetALD) [30]. Additionally, unhealthy sleep patterns and smoking habits are detrimental lifestyle factors that heighten the risk of MASH [31]. Disrupted or inadequate sleep has been linked to metabolic disturbances that promote insulin resistance and weight gain, whereas smoking intensifies oxidative stress and liver inflammation, further elevating the risk of metabolic syndrome and hepatic complications [32]. The interplay between dietary choices and lifestyle factors can significantly increase the likelihood of MASH development. For instance, a diet high in calories and fats coupled with a sedentary lifestyle result in more substantial weight gain and a heightened risk of metabolic syndrome, thereby creating an optimal environment for the onset of MASH.

### 2.2. Metabolic Factors of MASH

Central to the “multiple-hit” hypothesis is metabolic dysfunction, predominantly triggered by insulin resistance, which initiates an increase in lipogenesis and a decrease in fatty acid oxidation within the liver, leading to the continual accumulation of liver fat and modified lipid metabolism [6]. This issue is exacerbated by dysfunctional adipose tissue associated with obesity, which releases pro-inflammatory cytokines and inadequately retains lipids, thereby further augmenting the flux of free fatty acids to the liver [33]. Concurrently, molecular and cellular responses to stress intensify the condition. Oxidative stress emerges as mitochondria and peroxisomes, overwhelmed by fatty acids, produce an excess of ROS, inducing oxidative damage and malfunction in mitochondria. Additionally, the endoplasmic reticulum (ER) faces stress due to an abundance of unfolded proteins, triggering an unfolded protein response (UPR) that, if prolonged, leads to heightened cellular inflammation and cell death [34,35].

Hyperammonemia is a hallmark of advanced metabolic liver disease. Hyperammonemia occurs when liver dysfunction disrupts key urea cycle enzymes, such as carbamoyl phosphate synthetase 1 and ornithine transcarbamylase, reducing the liver’s ability to detoxify ammonia [36]. This situation is worsened by fatty liver infiltration, inflammation, and fibrosis, which decrease the number of functional hepatocytes and further impair urea cycle efficiency [36]. Together, these factors create a metabolic environment conducive to ammonia accumulation, exacerbating the systemic and hepatic complications of MASLD [37,38].

The immune reaction also plays a crucial role in the advancement of MASH. Kupffer cells, which are liver-residing macrophages, become activated in reaction to lipotoxicity and oxidative stress, releasing inflammatory cytokines and stimulating the NLRP3 inflammasome, which worsens liver inflammation and damage [39,40]. Furthermore, the liver encounters infiltration by immune cells such as neutrophils, monocytes, and lymphocytes, which respond to cytokines and chemokines from stressed hepatocytes and Kupffer cells, thereby intensifying the inflammatory environment [39,41]. Lastly, fibrogenesis is marked in the later stages of MASH, where activated hepatic stellate cells (HSCs), influenced by cytokines such as TGF-β and products of oxidative stress, transform into myofibroblasts that generate the overproduction of collagen and other extracellular matrix proteins [42]. This excessive matrix deposition leads to fibrosis, progressively altering the liver’s structure and resulting in cirrhosis [42]. Cirrhosis reduces liver function and aggravates the risk of developing hepatocellular carcinoma (HCC) along the course of disease [43]. Platelet-activating factor (PAF), a potent inflammatory mediator, has also been implicated in liver disease. Recent studies have demonstrated a link between PAF and its metabolic enzymes, such as Lp-PLA2, with gamma-glutamyl transferase, a well-known marker of liver disease [44]. PAF contributes to the pathogenesis of MASH by promoting hepatic lipid synthesis, which can result in hypertriglyceridemia. Notably, animal models have shown that knocking down the PAF receptor gene reduces inflammation and improves the glycemic profile, further supporting the role of PAF in metabolic dysfunction [45]. This thorough understanding of the multifaceted pathogenesis of MASH underscores the importance of addressing these interconnected pathways to develop effective interventions and treatments for the condition.

### 2.3. Genetic Factors of MASH

Along with metabolic factors, the occurrence of genetic factors is crucial in the development of MASLD and progression to MASH. Studies on human families have shown that children whose parents have elevated levels of fat in the liver are at a greater risk of developing and cirrhosis [46]. Genetic studies of human MASH have identified causative variants in genes such as PNPLA3, MBOAT7, TM6SF2, APOC3, and FATP5 are associated with lipid metabolism and accumulation [47]. The PNPLA3 gene variant rs738409 (C > G), which results in an I148M substitution, is strongly associated with increased hepatic fat content, inflammation, a risk of MASH, elevated liver enzymes, and fibrosis [7]. This variant is prevalent among various ethnic groups, particularly the Spanish population, and is also observed in lean MASLD patients [7,48]. The PNPLA3 variant leads to decreased lipid catabolism and increased lipid accumulation. The MBOAT7 gene variant rs641738 (C > T) is linked to increased liver fat content, enhanced de novo lipogenesis (DNL), and the progression of MASH [49]. This variant disrupts lipid homeostasis and promotes hepatic inflammation, with significant impacts observed in different ethnicities, particularly among European populations [49]. The TM6SF2 gene variant rs58542926 (C > T), which results in an E167K substitution, is associated with an increased risk of MASLD and fibrosis due to reduced VLDL secretion and lipid accumulation in the liver [7,48]. This variant is prevalent among lean MASLD patients and is linked to hepatic lipid aggregation and elevated ER stress [50]. The APOC3 gene variants rs2854116 and rs2070667 are associated with higher triglyceride levels, impaired lipid clearance, and increased susceptibility to MASLD and IR [51,52]. These variants inhibit lipoprotein lipase, increasing hepatic lipid uptake and promoting steatosis. The FATP5 gene variant rs56225452 is associated with increased hepatic steatosis, elevated ALT levels, and insulin resistance [47]. This gain-of-function variant enhances fatty acid uptake, contributing to MASLD pathogenesis [53].

Genetic variants related to cellular stress and the immune system are crucial in MASLD progression. The IRGM gene variants rs10065172 and rs13361189 impair lipophagy and mitophagy, leading to hepatic steatosis and inflammation [47,52]. These variants disrupt mitochondrial function and hepatic lipid metabolism, particularly in obese individuals [54,55]. The SOD2 gene variant rs4880 is associated with increased oxidative stress and advanced fibrosis [52,56]. This variant reduces enzymatic activity, leading to higher levels of ROS and promoting MASLD progression [57]. Inflammatory cytokines, including IL-32, TNF-α, IL-6, and IL-1β, have gene variants that enhance inflammation and the progression of MASLD to MASH and fibrosis [47]. Variants such as rs9788910 (IL-32), rs1800629 (TNF-α), rs1800795 (IL-6), and rs16944 (IL-1β) increase the expression and activity of these cytokines, contributing to hepatic inflammation and damage [33]. The HSD17B13 gene variant rs72613567 (T > TA) confers protection against MASLD, alcoholic liver disease, and hepatocellular carcinoma by reducing hepatic lipid biogenesis [47,52]. This loss-of-function splice variant is particularly beneficial for carriers of the PNPLA3 risk allele, mitigating liver damage [58]. Understanding these genetic factors is critical for developing precision medicine approaches to effectively diagnose, treat, and manage MASLD. Further research is essential to elucidate the molecular mechanisms of these variants and expand therapeutic options.

### 2.4. Epigenetic Factors

Epigenetic factors are increasingly acknowledged in the etiology of MASLD for their significant role in regulating gene expression while preserving the DNA sequence [59]. DNA methylation and chromatin remodeling are central mechanisms in these epigenetic processes. The DNA methylation of cytosine nucleotides at CpG-rich regulatory or promoter sites is vital in suppressing gene transcription related to metabolic and inflammatory pathways associated with MASLD [60]. Simultaneously, histone modifications like methylation and acetylation alter chromatin structure, affecting gene accessibility and expression levels [60]. Environmental influences such as dietary factors significantly impact epigenetic states, worsening disease phenotypes like MASLD [61]. For example, a high-fat diet (HFD) can induce epigenetic changes that enhance lipid accumulation and insulin resistance in hepatic tissues, key aspects of MASLD pathology. Studies of obese human females have demonstrated that the consequences of a poor fetal environment influence the risk of MASLD [62]. Another human study revealed that the maternal intake of fats impacts the advancement of MASLD in adult offspring [63]. This phenomenon is attributed to the impairment of mitochondrial metabolism in the liver and a rise in hepatic lipogenesis [64]. Other human studies demonstrate the correlation between intrauterine growth restriction and accelerated fetal growth with an increased susceptibility to the development of MASLD in later stages of life [65,66,67,68]. The exposure of a fetus to HFD can exacerbate liver injury by interfering with the cell cycle in liver cells via the activation of the p21 locus. This process is related to a prevailing inclination towards the demethylation of hepatic DNA, particularly at the p21 locus, which facilitates enhanced transcription and the onset of hepatocellular senescence [69]. On the other hand, DNA methylation alterations are also found to mediate the impact of aging on susceptibility to MASLD. Specifically, aging is associated with epigenetic modifications in hepatic DNA that disrupt mitochondrial energy metabolism and affect signaling and fibrogenic pathways [70]. Consistent with these findings, the increased methylation of PGC1a, which is critical for mitochondrial biogenesis, and of mitochondrial DNA that codes for enzymes essential for oxidative phosphorylation has been linked to reduced mitochondrial DNA content, insulin resistance, and the development of MASLD [71,72].

Recent studies in human subjects with various stages of MASH and mice models have underscored the crucial role of noncoding RNAs such as long noncoding RNAs (lncRNAs), microRNAs (miRNAs), and circular RNAs (circRNAs) in regulating gene transcription, particularly in conditions like diabetes [73,74], obesity [75], and MASLD [76,77]. LncRNAs, over 200 nucleotides long, modulate post-transcriptional processes like degradation, splicing, and translation while regulating epigenetics and gene transcription [78]. MiRNAs and circRNAs, part of the small noncoding RNA subgroup, are vital in controlling gene expression [79,80]. MiRNAs suppress gene expression by binding to the 3′-UTR of target mRNAs, while circRNAs sponge miRNAs to protect these mRNAs, a function that lncRNAs also perform [81]. Understanding the network of these noncoding RNAs is essential to unravel their impact on gene expression in MASLD. Thus, understanding epigenetic changes linked to MASLD is crucial for identifying novel therapeutic targets and developing effective treatments to manage or reverse the progression of MASH.

### 2.5. Microbiome in MASH

Alterations in gut microbiota composition have been consistently associated with MASLD and MASH [82,83]. Patients with MASH often exhibit an increased abundance of Proteobacteria and Enterobacteriaceae and a decreased presence of beneficial bacteria such as Bacteroidetes and Firmicutes [84,85]. These microbial shifts can influence metabolic and inflammatory pathways, promoting liver disease [86,87]. One of the key mechanisms involves the production and translocation of endotoxins, particularly lipopolysaccharides (LPS) [88]. The gut microbiome in MASLD/MASH patients often produces higher levels of endotoxins [5]. Due to an impaired intestinal barrier common in obesity and metabolic syndrome, these endotoxins translocate into the portal circulation, reaching the liver and triggering inflammatory responses [86]. The activation of toll-like receptor 4 (TLR4) on hepatic macrophages by LPS promotes the release of pro-inflammatory cytokines, exacerbating liver inflammation and fibrosis [88]. Bacterial metabolites are another critical aspect of the gut–liver axis in MASLD/MASH [89]. Metabolites such as trimethylamine N-oxide (TMAO) and imidazole propionate have been implicated in metabolic disturbances and liver pathology [90]. TMAO, derived from dietary choline and carnitine by gut microbiota, has been shown to aggravate hepatic steatosis and fibrosis by influencing bile acid metabolism and farnesoid X receptor (FXR) signaling [91,92]. Furthermore, TMAO is also well reported to contribute to endothelial dysfunction and increase the risk of cardiovascular complications [93,94,95]. Similarly, imidazole propionate, produced from histidine by gut bacteria, impairs insulin signaling and contributes to metabolic dysregulation [96].

The gut microbiota interacts with the host immune system, influencing the progression of MASLD/MASH [82]. Bacterial products, such as peptidoglycans and flagellin, can activate immune cells through pattern recognition receptors (PRRs), including TLRs and nucleotide-binding oligomerization domain (NOD)-like receptors [97,98]. This activation leads to the production of pro-inflammatory cytokines, such as TNF-α, IL-1β, and IL-6, which further drive hepatic inflammation and fibrosis [99,100]. Dietary factors significantly influence gut microbiota composition and function, thereby impacting MASLD/MASH development [101]. Diets high in fat and sugar, typical of Western dietary patterns, promote gut dysbiosis, characterized by an increase in harmful bacteria and a decrease in beneficial ones. This dysbiosis enhances the production of endotoxins and pro-inflammatory metabolites, contributing to liver disease. Conversely, diets rich in fiber and polyphenols can promote a healthy microbiota, potentially mitigating MASLD/MASH progression [101,102]. An impaired intestinal barrier is a hallmark of MASLD/MASH, facilitating the translocation of bacteria and their products into the liver [99]. Factors such as gut dysbiosis, high-fat diet, and chronic inflammation can disrupt the tight junctions in the intestinal epithelium, leading to increased intestinal permeability, often referred to as “leaky gut” [103]. This condition allows bacterial endotoxins and metabolites to enter the portal circulation and reach the liver, where they induce an inflammatory response [99,103,104]. A detailed understanding of the gut microbiome in MASLD and MASH pathogenesis through microbial changes, endotoxin production, and immune activation is pivotal for the development of potential therapies.

## 3. Therapeutic Targets in MASH

The multifactorial pathophysiological processes associated with MASH and MASLD necessitate the implementation of comprehensive therapeutic strategies for effective treatment. Targeting three key pathological domains, namely lipid metabolism and lipotoxicity, oxidative stress and inflammation, and insulin sensitivity and glucose homeostasis, offers a strategic approach to managing these complex diseases. Interventions modulating lipid metabolism are pivotal in reducing hepatic fat accumulation and preventing lipotoxicity, a significant driver of disease progression. Furthermore, therapies that mitigate oxidative stress and inflammation are critical in limiting hepatocellular damage and attenuating tissue fibrosis. Enhancing insulin sensitivity and regulating glucose homeostasis are equally essential for restoring metabolic balance and addressing the systemic metabolic dysregulation commonly associated with MASH and MASLD. By addressing these interconnected pathological mechanisms, current research and clinical efforts focused on therapeutic approaches can offer more targeted and effective treatment options for these complex liver diseases (summarized in Table 1).

### 3.1. Targeting Lipid Metabolism and Reducing Lipotoxicity

#### 3.1.1. Thyroid Hormone Receptor Beta Agonist

A pivotal aspect of managing MASH and MASLD involves regulating lipid metabolism to reduce lipotoxicity. One example are thyroid hormone receptor beta (THRβ) agonists, which regulate lipid metabolism to provide a comprehensive approach to lipid management [254]. THRβ agonists enhance fatty acid oxidation and reduce lipogenesis through the activation of the beta receptor in the liver, effectively decreasing lipid accumulation [255]. The key biological pathways involved in this process include the activation of AMP-activated protein kinase (AMPK) and the suppression of sterol regulatory element-binding protein-1c (SREBP-1c), which together promote lipid catabolism and inhibit lipid synthesis [256,257]. Moreover, these agonists improve mitochondrial function and increase the expression of genes involved in lipid transport and metabolism. These combined actions contribute significantly to their therapeutic effects in treating MASH [258].

Resmetirom, a selective THR-β agonist, treats MASH by enhancing fatty acid oxidation and reducing lipogenesis in the liver. It promotes mitochondrial biogenesis and mitophagy, thereby reducing lipid accumulation, inflammation, and fibrosis [133]. Additionally, resmetirom decreases triglyceride synthesis, promotes LDL cholesterol uptake, and enhances bile acid synthesis, leading to improved lipid profiles by upregulating deiodinase type 1 (DIO1), which improves intrahepatic thyroid hormone signaling and lipid metabolism, reducing hepatic lipotoxicity [133,134]. Clinical studies have demonstrated that resmetirom significantly reduces hepatic fat content, liver enzymes, fibrosis markers, and cardiovascular risk factors. Specifically, the efficacy of resmetirom was assessed in a 36-week, phase 2 trial (NCT02912260) involving patients with biopsy-confirmed MASH (fibrosis stages 1–3; NAS ≥ 4; and hepatic fat fraction ≥ 10%) who received either 80 mg of resmetirom or a placebo daily [133,135,136]. The primary endpoint was the change in hepatic fat content at 12 weeks, with secondary endpoints including fat reduction at 36 weeks, liver biopsy results, and improvements in liver enzymes and lipid profiles [135]. Resmetirom significantly reduced hepatic fat, liver enzymes, and atherogenic lipids compared to the placebo, with mild side effects like diarrhea and nausea [133,135]. The study concluded that early fat reduction predicted MASH resolution and fibrosis improvement, highlighting resmetirom’s potential efficacy [133,135].

The phase 3 MAESTRO-NAFLD-1 trial (NCT04197479) assessed the safety and efficacy of resmetirom in 1143 obese patients with MASLD and presumed MASH who did not meet the histological criteria for MAESTRO-NASH [137]. Participants received 80 mg or 100 mg of resmetirom or a placebo daily for 52 weeks [138]. The primary endpoint was the safety profile of resmetirom, which was confirmed to be acceptable, with common mild to moderate gastrointestinal issues such as diarrhea and nausea [139]. Serious adverse events were similar between the resmetirom and placebo groups, including gastrointestinal disorders, gallstone-related issues, cardiac disorders, respiratory infections, musculoskeletal disorders, and nervous system disorders [139]. There were no major adverse cardiovascular events, increases in bone fractures, or significant changes in bone mineral density T-scores [139]. Secondary outcomes showed significant reductions in hepatic fat content, with decreases of 34.9% and 38.6% at Week 16 and 28.8% and 33.9% at Week 52 for the 80 mg and 100 mg groups, respectively [139]. The trial also observed significant reductions in liver stiffness, as measured by fibroscan, in both resmetirom groups compared to the placebo [139]. The phase 3 MAESTRO-NASH trial (NCT03900429) evaluated resmetirom (MGL-3196) in 966 obese adults with biopsy-confirmed MASH and fibrosis stages 1–3. Participants received 80 mg or 100 mg of resmetirom or a placebo daily for 52 weeks [140,141]. The primary endpoints were the resolution of MASH without worsening fibrosis and an improvement in fibrosis by at least one stage. MASH resolution occurred in 25.9% (80 mg) and 29.9% (100 mg) of participants, compared to 9.7% in the placebo group [141]. Fibrosis improvement was seen in 24.2% (80 mg) and 25.9% (100 mg) of participants versus 14.2% in the placebo group. Both co-primary endpoints were met by 14.2% (80 mg) and 16.0% (100 mg) of participants, compared to 4.9% of the placebo group [141]. The secondary outcomes included significant reductions in hepatic fat content, with decreases of 34.9% and 38.6% at Week 16 and 28.8% and 33.9% at Week 52 for the 80 mg and 100 mg groups, respectively [141]. Additionally, there were significant reductions in serum liver enzymes and improvements in non-invasive fibrosis biomarkers such as ELF and PRO-C3 [141]. Overall, the phase 3 MAESTRO-NASH and MAESTRO-MASLD-1 trials highlight the potential of resmetirom as a safe and effective treatment for MASH and MASLD [134]. The trials’ positive outcomes, coupled with the urgent need for effective therapies, led to the accelerated approval of resmetirom by the U.S. Food and Drug Administration (FDA) on 14 March 2024 [134]. This landmark decision makes resmetirom the first-ever FDA-approved treatment for MASH [134].

#### 3.1.2. Acetyl-CoA Carboxylase Inhibitors

Acetyl-CoA carboxylase inhibitors (ACC inhibitors) and diacylglycerol acyltransferase 2 inhibitors (DGAT2 inhibitors) are critical, as they inhibit key enzymes in fatty acid and triglyceride synthesis, respectively. In recent works by Calle et al. [105] and Zhang et al. [108], innovative approaches were used to investigate the efficacy of ACC in MASH treatment. These studies effectively addressed hyperlipidemia side effects while demonstrating strong anti-MASH effects. Two concurrent phase 2a trials delved into the liver-targeted inhibition of ACC1/2 in adults with MASLD. The initial trial (NCT03248882) [106] assessed the novel ACC1/2 blocker PF-05221304 at varying doses (2, 10, 25, and 50 mg once daily) versus a placebo for 16 weeks, while the second trial (NCT03776175) [107] investigated PF-05221304 (15 mg twice daily) in conjunction with the DGAT2 blocker PF-06865571 (300 mg twice daily) versus a placebo for 6 weeks. Both trials measured the change in liver fat percentage using MRI proton density fat fraction. Monotherapy with PF-05221304 exhibited a dose-dependent reduction in liver fat, achieving reductions of 50–65% at doses of 10 mg or higher [105]. Adverse events (AEs) did not escalate with increased doses, except for a rise in serum triglycerides in 8% of patients which is a known side effect, leading to withdrawal in 4% of patients. The combined therapy notably decreased liver fat compared to the placebo, surpassing the efficacy of each drug independently [105]. While 36% of patients on the combined regimen reported AEs, no discontinuations were attributed to AEs. Furthermore, the combination mitigated the impact of the ACC inhibitor on serum triglycerides, suggesting that the co-administration of PF-05221304 and PF-06865571 could address certain limitations of ACC inhibition in isolation [105]. In another study, researchers identified a small molecule, IMA-1, that disrupts the interaction between arachidonate 12-lipoxygenase (ALOX12) and acetyl-CoA carboxylase 1 (ACC1), offering a novel approach to MASH treatment [108]. IMA-1 demonstrated remarkable efficacy in halting diet-induced MASH progression in both male mice and cynomolgus macaque models, with results comparable to those of ACC inhibitors. Protein docking simulations and functional experiments revealed that IMA-1’s anti-MASH effects stem from direct binding to a specific pocket in ALOX12 near its ACC1-interacting surface, without inhibiting ALOX12’s lipoxygenase activity, thus avoiding common side effects. Notably, IMA-1 did not induce hyperlipidemia, a frequent adverse effect of direct ACC inhibition, in both mice and macaques. These findings suggest the therapeutic potential of IMA-1 as a small-molecule treatment for NASH, highlighting the promising future of such therapies in managing and treating NASH across multiple species [108].

#### 3.1.3. Diacylglycerol Acyltransferase 2 Inhibitor

Another clinical study evaluated the histological outcomes following the oral administration of a DGAT2 inhibitor and its combination with an acetyl-coenzyme A carboxylase inhibitor in participants with biopsy-confirmed MASH and fibrosis in stages F2 or F3 [259]. The study employed a meticulous triage approach, incorporating double-confirmation with non-invasive blood tests and quantitative ultrasound markers before the baseline liver biopsy and central readings of all biopsies to enhance participant selection and ensure accurate histological findings. Throughout the dosing period, the study conducted longitudinal evaluations using non-invasive imaging and blood-based biomarkers to correlate with histological changes and assess drug effects. Bayesian dose–response modeling was used to efficiently characterize dose–response relationships and inform phase 3 dose selection. While the results were promising, the study’s reliance on translating effects from earlier non-biopsy studies to histological endpoints necessitates further confirmation of the drug’s impact on clinical outcomes in a larger phase 3 trial [259].

#### 3.1.4. Fibroblast Growth Factor Analogs

Fibroblast growth factor analogs, specifically FGF19 and FGF21, play significant roles in modulating bile acid synthesis and lipid metabolism while also enhancing glucose metabolism [260]. FGF19, primarily produced in the ileum, is released postprandially and regulates bile acid metabolism by inhibiting CYP7A1, thereby reducing bile acid production in the liver. It signals through FGFR4 and β-Klotho (KLB), leading to reduced liver fat and fibrosis [260]. On the other hand, FGF21, produced in the liver, pancreas, muscle, and adipose tissue, acts endocrinologically via FGFR1c, FGFR2, and FGFR3 with KLB [260]. It regulates glucose and lipid metabolism, promotes insulin sensitivity, induces weight loss, and reduces hepatic triglycerides by inhibiting lipogenic gene expression and enhancing lipid utilization [260]. FGF21 also attenuates hepatic fibrogenesis by downregulating the TGF-β and NF-κB pathways, making it a promising candidate for NASH treatment [260].

FGF19 and FGF21 analogs are emerging as promising treatments for MASH, showing significant efficacy in clinical trials [261,262]. Aldafermin (NGM282), an FGF19 analog, has demonstrated remarkable improvements in liver fat content and fibrosis markers [109]. In a phase 2 study, 68% of aldafermin-treated patients achieved a ≥5% reduction in liver fat, compared to just 24% in the placebo group, with 38% showing fibrosis improvement without worsening MASH [109]. Efruxifermin (EFX, also known as AKR-001 or AMG876) is a genetically engineered Fc-FGF21 fusion protein with improved pharmacokinetics and pharmacodynamics. It is currently in phase 2 clinical trials for MASH, fibrosis, and compensated liver cirrhosis. EFX has shown potential in improving glycemic control, has a good safety profile, and demonstrates antifibrotic effects, meeting FDA criteria for phase 3 trials [110]. Similarly, FGF21 analogs, BFKB8488A, have proven effective in reducing liver fat and enhancing metabolic parameters [111]. In a phase 1 trial, BFKB8488A significantly decreased the hepatic fat fraction and improved lipid profiles, maintaining a favorable safety profile. This study highlighted that a long-acting FGF21 analog, BFKB8488A, significantly reduced body weight, fasting triglycerides, and LDL cholesterol while increasing HDL cholesterol and adiponectin levels [111].

#### 3.1.5. Farnesoid X Receptor Agonist

Farnesoid X receptor (FXR) plays a pivotal role in the pathogenesis of MASH by modulating several critical pathways, including bile acid synthesis and enterohepatic circulation, lipid and glucose metabolism, inflammation, fibrosis, gut barrier integrity, and intestinal microbiota [112]. The therapeutic impacts of FXR in MASH are mediated through several pathways. Specifically, FXR controls bile acid synthesis and homeostasis by inhibiting CYP7A1 via the activation of the small heterodimer partner (SHP) and by enhancing FGF19, which interacts with the FGFR4 receptor in hepatocytes [262,263,264]. In lipid metabolism, FXR downregulates sterol regulatory element-binding protein-1c (SREBP-1c), thereby reducing lipogenesis, and activates peroxisome proliferator-activated receptor alpha (PPARα), leading to increased fatty acid oxidation [265]. Regarding glucose metabolism, FXR inhibits phosphoenolpyruvate carboxykinase (PEPCK) and glucose-6-phosphatase (G6Pase), resulting in decreased gluconeogenesis and improved peripheral insulin sensitivity [266]. FXR’s anti-inflammatory effects are achieved through the inhibition of the nuclear factor kappa-light-chain-enhancer of activated B cells (NF-κB) and the NLRP3 inflammasome, leading to reduced pro-inflammatory cytokines and hepatocellular damage. In terms of fibrosis reduction, FXR inhibits the transforming growth factor beta (TGF-β) pathway, leading to decreased activation in hepatic stellate cells and collagen synthesis [267,268,269]. Additionally, FXR reinforces gut barrier integrity and regulates microbiota composition by augmenting antimicrobial peptides and promoting a healthy gut microbiome [270]. The activation of the FGF19/FGFR4 pathway by FXR further manages bile acid synthesis and enhances systemic metabolic effects, such as reduced fat accumulation and improved glucose balance. These interconnected pathways underscore the crucial role of FXR in regulating the metabolic, inflammatory, and fibrotic mechanisms associated with MASH.

Obeticholic acid (OCA), also known as INT-747, is a potent and selective FXR agonist derived from chenodeoxycholic acid (CDCA), a natural bile acid [113]. By activating FXR in the liver and intestines, OCA regulates several metabolic pathways, including inhibiting bile acid synthesis via CYP7A1 suppression [114]. This reduces hepatic bile acid levels and promotes their excretion. Additionally, OCA reduces lipogenesis, decreases hepatic triglyceride accumulation, improves insulin sensitivity, and has anti-inflammatory and antifibrotic effects, making it a promising candidate for MASH treatment [115]. Preclinical studies have shown that OCA lowers liver fat, inflammation, and fibrosis in animal models [115]. The subsequent phase 2b clinical FLINT trial (NCT01265498) confirmed therapeutic benefits in MASH patients with significant improvements in liver histology despite side effects like pruritus and altered lipid profiles [112,116]. Initially approved for primary biliary cholangitis under the brand name Ocaliva, current research aims to optimize its use, potentially in combination with other agents, to enhance its efficacy while minimizing side effects [117]. The phase 3 REGENERATE trial (NCT02548351) further evaluated its effectiveness, showing improved fibrosis in 23% of patients, though it did not meet the endpoint for MASH resolution, leading to more investigations on OCA’s long-term efficacy and safety before approval for MASH [118,119].

Tropifexor (LJN-452) is a potent, non-steroidal, selective FXR agonist with an EC_50_ value of 0.2 nM, with preclinical studies demonstrating its efficacy in two mouse models of NASH, where it significantly reduced steatohepatitis, decreased hepatic triglycerides, and reversed established liver fibrosis [120]. Tropifexor showed a gene expression profile indicating reduced oxidative stress, fibrogenesis, and inflammation, with efficacy at doses below 1 mg/kg, superior to the effects observed with OCA at 25 mg/kg in the same models [120]. Phase I trials showed dose-dependent target engagement in healthy volunteers without affecting plasma lipids [121]. The phase 2 FLIGHT-FXR trial (NCT02855164) assessed its impact in MASH patients, revealing significant reductions in ALT and GGT levels, body weight, and the MRI-estimated proton density fat fraction (MRI-PDFF) over 48 weeks [122,123]. However, Tropifexor did not show significant differences in the NAFLD Activity Score (NAS), MASH resolution, or fibrosis compared to a placebo as measured by conventional microscopy. Post hoc digital analysis showed reductions in overall liver fibrosis and the regression of perisinusoidal fibrosis in patients with advanced fibrosis at baseline [123]. The study also highlighted a high incidence of pruritus and changes in lipid profiles, such as increased LDL-C and decreased HDL-C, especially at higher doses [123]. In the TANDEM trial (NCT03517540), LJN-452 with cenicriviroc, a dual CCR2/CCR5 antagonist, was used to study patients with biopsy-proven NASH and liver fibrosis [124,271]. While combination therapy showed comparable safety profiles to monotherapy, no substantial improvement over Tropifexor alone was observed [124].

Another non-steroidal FXR agonist for treating MASH and other liver diseases is Cilofexor (GS-9674) [125,126]. Its primary function is to activate FXR, which helps reduce liver fat, inflammation, and fibrosis by regulating bile acid synthesis and promoting lipid metabolism [125]. Preclinical studies demonstrated that Cilofexor decreases portal hypertension in cirrhotic and non-cirrhotic models of MASH by targeting vascular remodeling and sinusoidal dysfunction without affecting systemic hemodynamics [127]. Additionally, Cilofexor was shown to reduce liver fibrosis and intrahepatic sinusoidal resistance, contributing to lowered portal hypertension via the deactivation of hepatic stellate cells and inhibition of fibrogenesis [126]. In a phase 2 clinical trial, a 24-week treatment with Cilofexor in patients with non-cirrhotic MASH showed a significant reduction in hepatic steatosis and improved liver biochemistry [128]. However, the changes were modest compared to those with OCA [128]. While serum markers of fibrosis were reduced, there were no significant changes observed in serum lipid profiles [128]. Increased pruritus was seen in patients who received higher doses of Cilofexor (100 mg) [128]. Cilofexor is also being explored in combination with other therapies to enhance their efficacy. For example, Cilofexor was combined with the ACC inhibitor firsocostat and showed promising results in reducing liver transaminases, bile acids, and fibrosis markers in patients with MASH [129].

Vonafexor (EYP001), is another non-steroidal FXR agonist designed to selectively activate FXR without interacting with TGR5. Vonafexor has shown efficacy comparable to OCA in preclinical studies [130]. In animal models, Vonafexor demonstrated significantly improved MASH-related parameters, including fibrosis, ballooning, and portal inflammation [130]. Based on these positive preclinical results and promising data from phase 1 clinical trials regarding its safety, tolerability, and pharmacodynamics, Vonafexor advanced into phase 2 trials for NASH treatment [131]. In a 12-week phase 2 clinical trial (NCT03812029), Vonafexor significantly reduced liver fat content measured by MRI-PDFF, reduced body weight and liver enzymes such as ALT and GGT, and improved creatinine-based glomerular filtration rates (GFRs) [131,132]. However, some patients experienced mild to moderate pruritus, which was more frequent at higher doses [131]. Despite these side effects, the efficacy and safety profile of Vonafexor makes it a valuable candidate in ongoing NASH research.

Given its multifaceted impact on these processes, FXR has emerged as a significant pharmacological target in NASH treatment, evidenced by the diversity of FXR agonists currently undergoing clinical trials [272]. These investigational compounds are bile acid derivatives, non-bile acid-derived steroidal FXR agonists, non-steroidal FXR agonists, and partial FXR agonists with class-specific effects, including pruritus, imbalances in low-density and high-density lipoproteins, and modest increases in alkaline phosphatase levels. However, their therapeutic potential in MASH is underscored by their clinically validated antifibrotic effects, which support their use either as monotherapy or in combination with other treatments.

#### 3.1.6. Peroxisome Proliferator-Activated Receptors

Peroxisome proliferator-activated receptors (PPARs) are nuclear receptors activated by specific agonists, including natural fatty acid derivatives and synthetic drugs. They play a critical role in regulating whole-body lipid and glucose metabolism and inflammation [273,274]. The three PPAR family members—α, β/δ, and γ—exhibit both inter-organ and intra-organ differential expression patterns, leading to complementary activities in pathways implicated in the pathogenesis of MASH [275]. PPARα, mainly expressed in the liver, muscles, and kidneys, enhances fatty acid oxidation and improves lipid profiles by upregulating β-oxidation pathways while also increasing insulin sensitivity and exhibiting anti-inflammatory effects by inhibiting NF-κB signaling [276,277,278]. PPARβ/δ, ubiquitously expressed, promotes fatty acid oxidation and energy expenditure in muscle and adipose tissue, enhances glucose uptake and utilization, and modulates inflammation by reducing pro-inflammatory cytokines through the inhibition of the NF-κB and AP-1 pathways [279]. PPARγ, primarily found in adipose tissue, regulates adipogenesis and lipid storage by upregulating genes involved in fatty acid uptake and storage, improves insulin sensitivity and glucose metabolism by promoting glucose transport and adiponectin secretion, and exerts anti-inflammatory effects by suppressing pro-inflammatory genes and inhibiting macrophage activation [280,281]. Together, these PPARs provide a comprehensive approach to managing lipid and glucose homeostasis and reducing inflammation, making them key targets for treating metabolic disorders such as MASLD and MASH.

Extensive preclinical evidence demonstrating anti-MASH activity has prompted the clinical testing of various PPAR-modulating drugs for MASH treatment [275,282]. While several single and dual PPAR agonists have shown some efficacy in individual histological parameters, pan-PPAR agonism seems necessary to achieve significant improvements in histological endpoints. Lanifibranor, a pan-PPAR agonist, was tested in a phase 2b trial with 247 patients with highly active MASH, showing significant liver histology improvements without worsening fibrosis [142,143]; a phase 3 trial (NCT04849728) with 1000 participants is ongoing to confirm these findings [144]. Elafibranor, a dual PPARα/δ agonist, produced positive results in a phase 2 trial (NCT01694849) for improving MASH-related histological parameters [145], but the phase 3 trial (NCT02704403) failed to meet the primary endpoint of MASH resolution without fibrosis progression, leading to its discontinuation for MASH treatment [146]. Saroglitazar, a PPARα/γ dual agonist, improved liver enzyme levels, hepatic fat content, and metabolic parameters in phase 2 trials (NCT03061721) [147] and is approved in India for MASH and pending broader international approval [148]. Pemafibrate, a selective PPARα agonist, showed benefits in hepatic inflammation, fibrosis, and biochemical scores in preclinical studies [149,150] and a phase 2 trial (NCT03350165) in Japan, though further trials are needed to fully establish its efficacy and safety [151]. Overall, while some PPAR agonists show potential for treating MASLD/MASH, their varied pharmacokinetic and pharmacodynamic properties require individualized clinical evaluations to ensure their efficacy and safety. PPARs also modulate intra- and extrahepatic vascular dysfunction, which contributes to hepatic and cardiovascular morbidity in MASH [283]. Each PPAR agonist is chemically distinct, resulting in different pharmacokinetic and pharmacodynamic properties, and thus necessitates individual efficacy and safety assessments.

#### 3.1.7. Stearoyl-CoA Desaturase 1 Modulators

Stearoyl-CoA desaturase 1 (SCD1) modulators also reduce monounsaturated fatty acid synthesis, mitigating lipotoxic effects [284]. SCD1 is a key enzyme in the biosynthesis of monounsaturated fatty acids, which are crucial substrates for de novo lipogenesis, leading to increased triglyceride production and accumulation in the liver. Experimental studies have demonstrated that the liver-specific inhibition of SCD1 attenuates the development of hepatic steatosis and MASLD [285,286,287,288]. A recent study showed that inhibiting SCD1 with antisense oligonucleotides increased fatty acid oxidation and reduced de novo fatty acid synthesis, alleviating steatosis in hepatocyte cell lines and mouse models [289]. MicroRNAs like miR-103, miR-212-5p, and miR-27a were found to suppress SCD1 and fatty acid synthase (FAS), reducing diet-induced obesity and hepatic lipid accumulation [290,291,292]. Novel SCD1 inhibitors, such as N-(2-hydroxy-2-phenylethyl)-6-[4-(2-methylbenzoyl) piperidin-1-yl] pyridazine-3-carboxamide, reduced hepatic lipid accumulation and inflammation in MASH rat models [293]. Another selective inhibitor, 3-[4-(2-chloro-5-fluorophenoxy)-1-piperidinyl]-6-(5-methyl-1,3,4-oxadiazol-2-yl)-pyridazine, decreased triglyceride accumulation and enhanced liver functions in human stem cells [294]. Another study identified thiazole-4-acetic acid analog 48, a liver-specific SCD1 inhibitor with anti-diabetic and anti-obesity effects in rodents [295]. In Zucker fatty rats, the SCD1 inhibitor GSK993 reduced hepatic lipids and improved glucose tolerance and insulin sensitivity [296]. Additionally, dexmedetomidine (DEX) improved insulin sensitivity and reduced hepatic steatosis and inflammation in MASLD mice by lowering SCD1 levels [152]. These findings indicate that SCD1 inhibition is a promising therapeutic strategy for controlling and managing MASLD.

#### 3.1.8. Antisense Oligonucleotides Targeting Lipid Metabolism

Antisense oligonucleotides (ASOs) targeting PNPLA3, also known as AZD2693 or ION839, targets the PNPLA3 gene, specifically the I148M variant linked to increased triglyceride accumulation in the liver [153]. By reducing the expression of this mutated protein, ASOs effectively lower hepatic lipid storage and mitigate liver damage. Pre-clinical studies have shown that silencing PNPLA3 in animal models significantly reduced liver triglyceride levels and alleviated steatosis, with pronounced effects in those carrying the I148M variant, which impairs triglyceride degradation [297]. ASOs are being developed as a targeted therapy for NASH patients with the PNPLA3 I148M variant, addressing a key genetic risk factor in disease progression [153]. Currently, AZD2693 is in phase 2 clinical trials (NCT05809934) to evaluate its safety and efficacy in reducing liver fat and fibrosis in this genetically predisposed population [154].

The DGAT2 ASO, also known as ION224, targets the enzyme DGAT2 that is essential for the final step of triglyceride synthesis [155]. By inhibiting DGAT2, this ASO reduces the conversion of diacylglycerol and fatty acyl CoA into triglycerides, thereby decreasing hepatic lipid accumulation [156]. Pre-clinical studies have demonstrated that the DGAT2 ASO significantly lowers hepatic triglyceride content and improves liver histology in various animal models, including those fed high-fat diets and genetically obese mice, with reductions in liver inflammation and fibrosis [157,158]. ION224 is being developed as a treatment for MASH by addressing one of its core pathogenic mechanisms of triglyceride overload in hepatocytes. Phase 2 clinical trials (NCT04932512) in NASH patients have been completed, showing improvements in liver histology without worsening fibrosis, supporting its potential as a therapeutic option for reducing liver fat and inflammation in MASH [159,160].

High levels of serine/threonine protein kinase 25 (STK25) are associated with increased liver fat accumulation and reduced β-oxidation, contributing to the development of MASLD and MASH [161]. STK25 ASO targets STK25, a regulator of lipid metabolism and insulin sensitivity [162]. It works by reducing the activity of STK25, which is involved in the regulation of lipid partitioning in hepatocytes [162]. In pre-clinical studies, the genetic disruption or antisense-mediated knockdown of STK25 in animal models has shown significant reductions in liver steatosis and improved insulin sensitivity. Currently, the STK25 ASO is in the pre-clinical development stage, with research focusing on optimizing its safety and efficacy before progressing to clinical trials [163].

Loss-of-function variants of HSD17B13, such as rs72613567, have been associated with a reduced risk of chronic liver diseases, including MASLD, MASH, and HCC [47,52]. These variants result in truncated or unstable proteins with reduced enzymatic activity, providing a protective effect against liver damage [52]. Studies have shown that individuals with these genetic variants exhibit lower liver enzyme levels and less liver fat accumulation, suggesting a significant role in mitigating disease progression [47]. Therapeutic oligonucleotides targeting HSD17B13 aim to replicate the protective effects of these genetic variants by silencing the gene’s expression, thereby reducing inflammation and fibrosis in MASH patients [298]. Early clinical trials, such as those with ARO-HSD, are investigating the safety and efficacy of these approaches, with promising initial results in reducing hepatic HSD17B13 mRNA expression and protein levels (NCT04202354, NCT05143905, NCT05560607) [164,165,166,167].

### 3.2. Reducing Oxidative Stress and Inflammation

#### 3.2.1. Apoptosis Signal-Regulating Kinase 1

Oxidative stress and inflammation are among the critical drivers of MASH progression via apoptosis [299]. Apoptosis is a key pathway in hepatocellular death in MASLD, particularly in patients with abdominal obesity or insulin resistance [300]. Increased free fatty acid influx to the liver and enhanced hepatic lipogenesis lead to hepatic steatosis [300]. This steatosis causes lipotoxicity, which induces oxidative and endoplasmic reticulum stress, resulting in the accumulation of unfolded proteins [300]. Additionally, patients with MASH often experience small intestinal bacterial overgrowth, producing endotoxins and cytokines like TNFα, which contribute to hepatic inflammation and necrosis [301]. Apoptosis, driven by mediators such as CHOP, PP1 activator, GADD34, JNK, and ASK1, plays a crucial role in inflammation and fibrosis in MASLD [300]. Recent studies also highlight necroptosis, a form of cell death activated by the necrosome, as significant in MASLD progression [300]. ASK1 is crucial in the development of MASH by mediating stress-induced apoptosis and inflammation [302]. Various stressors, such as saturated free fatty acids, oxidative stress, and inflammatory cytokines like TNFα, activate ASK1. Upon activation, ASK1 initiates the JNK and p38 MAPK pathways, resulting in hepatocyte apoptosis, inflammation, and fibrosis [302]. ASK1 phosphorylates MKK4 and MKK7, which activate JNK1, worsening insulin resistance and hepatic steatosis [302]. Additionally, ASK1 phosphorylates MKK3 and MKK6, leading to p38 MAPK activation, further promoting inflammation and apoptosis [302]. Studies have shown that inhibiting ASK1 can reduce hepatic macrophage and stellate cell activation, thereby decreasing hepatic inflammation and fibrosis [302]. Animal models and early human trials indicate that ASK1 inhibition holds promise as a therapeutic strategy for MASH.

In preclinical studies, ASK1-deficient mice showed reduced weight gain, less visceral fat, and smaller increases in insulin resistance when fed a high-fat diet compared to wild-type mice [303]. These mice also exhibited less severe hepatic steatosis and fibrosis, indicating the protective effects of ASK1 inhibition against diet-induced liver damage [303]. Furthermore, mice overexpressing a constitutively active form of ASK1 in the liver developed more pronounced insulin resistance and hepatic steatosis under a high-fat diet, highlighting ASK1’s role in promoting these conditions [303]. In human studies, a phase 2 trial involving 242 MASH patients with stage 2 or 3 fibrosis assessed the efficacy of selonsertib, an oral ASK1 inhibitor [168]. Patients treated with selonsertib showed a significant reduction in fibrosis and serum markers of apoptosis and necrosis, along with improved liver enzyme levels [168]. The treatment was generally well-tolerated, although some patients experienced sinusitis and nasopharyngitis more frequently [168]. Phase 3 trials of selonsertib for patients with MASH and either bridging fibrosis (STELLAR-3, NCT03053050) or compensated cirrhosis (STELLAR-4, NCT03053063) were initiated, but both studies were terminated early due to insufficient efficacy [169,170].

#### 3.2.2. C-C Chemokine Receptor Antagonists

C-C chemokine receptor (CCR) antagonists, particularly those targeting CCR2 and CCR5, offer significant therapeutic potential in the treatment of MASH by addressing key mechanisms of hepatic inflammation and fibrosis [304]. CCR2, expressed in monocytes and macrophages, facilitates their recruitment into the liver via ligands such as CCL2 (MCP-1), leading to Kupffer cell activation and a subsequent inflammatory response [305]. Inhibiting CCR2 reduces the influx and activation of these immune cells, thereby mitigating hepatic inflammation [306,307]. CCR5, present in macrophages, T-cells, and hepatic stellate cells (HSCs), promotes immune cell infiltration and HSC activation through ligands like CCL5 (RANTES—regulated on activation, normal T cell expressed and secreted), contributing to fibrogenesis. CCR5 antagonism disrupts these processes, diminishing both inflammation and fibrosis [308]. Cenicriviroc (CVC), a dual CCR2/CCR5 antagonist, exemplifies the efficacy of this approach by simultaneously reducing pro-inflammatory monocyte/macrophage activity and HSC-mediated fibrogenesis [171,172], as evidenced by clinical trials such as the CENTAUR phase 2b trial (NCT02217475), which demonstrated significant improvements in liver fibrosis and markers of liver injury [173]. Thus, targeting CCR2 and CCR5 pathways with specific antagonists provides a promising strategy for alleviating the pathological features of MASH.

#### 3.2.3. Galectin-3 Inhibitors

Galectin-3 is crucial in fibrogenesis and inflammatory responses, primarily through its interaction with TGF-β1, a key cytokine that activates HSCs into myofibroblasts, leading to extracellular matrix deposition and fibrosis. Galectin-3 enhances TGF-β1 signaling by forming the “galectin-3 fibrosome”, which cross-links TGF-β1 receptors and integrins on the cell surface, facilitating fibrotic pathways [309]. Additionally, galectin-3 interacts with the CD98hc and β1-integrin complex, mediating inflammatory responses, thus playing a central role in the fibrotic processes of liver diseases like MASLD and MASH [310]. Galectin-3 inhibitors, encompassing small molecules and larger polysaccharides, target the extracellular activity of galectin-3 by binding to its CRD and, in some cases, its N-terminal domain [311,312]. By disrupting these interactions, galectin-3 inhibitors prevent the formation of oligomeric structures and lattice-like assemblies that are essential for activating pro-fibrotic signaling pathways, thereby halting fibrosis and reducing liver inflammation in MASLD patients [313]. Consequently, galectin-3 inhibitors offer a novel therapeutic strategy for mitigating fibrosis and inflammation in MASLD and preventing its progression to more severe liver diseases.

Initial studies to identify the anti-inflammatory and anti-fibrotic activities of galectin-3 were conducted using HSCs, macrophages, and animal models of liver disease [314,315]. In vitro studies on HSCs demonstrated that galectin-3 is critical for their activation into myofibroblasts, central to the fibrotic process, elucidating the mechanisms by which galectin-3 facilitates extracellular matrix deposition [316]. In vivo studies, particularly in rodent models, used mice with galectin-3 knockout to show that the absence of this protein significantly reduces fibrosis in response to liver injury, providing compelling evidence of its anti-fibrotic potential [316,317]. Additionally, studies on macrophages highlighted galectin-3’s role in mediating inflammatory responses, as macrophages from galectin-3-deficient mice exhibited reduced inflammatory cytokine production [318,319,320].

Belapectin, a large-molecule polysaccharide inhibitor derived from natural sources, showed promise in a phase 2 clinical trial for preventing esophageal varices in patients with compensated cirrhosis due to NASH [174]. This trial revealed that belapectin could reduce the hepatic venous pressure gradient, suggesting a beneficial impact on liver function and structure [174]. Following these results, an international adaptive phase 2b/3 trial (NCT04365868) was initiated to further investigate its clinical benefits in preventing esophageal varices based on endoscopic evaluations [175]. GB1107, a small-molecule thiogalactoside inhibitor targeting the CRD of galectin-3, is also under investigation [176,177]. GB1107’s analog, GB1211, is being evaluated in a phase 2 study (NCT04607655) for cirrhosis across various etiologies in a first-in-human trial [178].

#### 3.2.4. RXFP1 Agonists

Relaxin is a well-known reproductive hormone that plays an important role in maternal adaptations to pregnancy by acting on its cognate receptor, relaxin/insulin-like family peptide receptor 1 (RXFP1) [179,180]. However, pleiotropic effects of relaxin, which include vasoprotective activities, anti-fibrotic and anti-inflammatory actions, have been reported in non-reproductive organs [181,182,183,184]. This has led to the development of relaxin-based therapeutics for the treatment of several cardiometabolic diseases, including MASLD and MASH [185,186,187,188]. Indeed, relaxin is reported to have had beneficial effects in several preclinical animal models of chronic liver injury/disease. Specifically, the activation of RXFP1 reduced fibrosis in models of progressive and established hepatic fibrosis with (high-fat-diet model) or without insulin resistance (methionine–choline-deficient-diet, bile duct ligation, or carbon tetrachloride model) [189,190,191,192,193], which was in part mediated by changes to gene signatures related to extracellular matrix remodeling and cytokine signaling [192,193]. Furthermore, Hu et al. used lipid nanoparticles combined with aminoethyl anisamide, a powerful agonist for sigma-1 receptor, which is abundant in activated HSCs, to facilitate hepatic transport of the relaxin gene in mice. This innovative gene delivery method caused a selective increase in relaxin levels, which resulted in a significant inhibition of fibrosis [194]. Particularly, it demonstrated the transformation of pro-fibrotic hepatic macrophages into regenerative phenotypes responsible for coordinating the process of tissue restoration in two dietary models of MASH [194]. Moreover, relaxin administration also ameliorated renal dysfunction in patients with cirrhosis and portal hypertension (NCT01640964) while maintaining the safety profile and pharmacokinetics (NCT02669875); thus, no dose adjustment is needed for relaxin treatment in patients with hepatic impairment [195,196]. In addition to anti-fibrotic and anti-inflammatory effects in the liver, many studies have also reported that relaxin improved vascular endothelial function in preclinical models of cardiometabolic diseases and clinical settings [197,198,199,200]. Indeed, relaxin treatment prevented endothelial dysfunction in renal arteries isolated from animal models of liver disease and improved renal blood flow in cirrhotic patients [190,195]. Taken together, the pleiotropic effects of relaxin suggest that it may have enormous therapeutic potential for the treatment of liver fibrosis and vascular dysfunction associated with MASH.

#### 3.2.5. L-Ornithine L-Aspartate

L-ornithine L-aspartate (LOLA) has emerged as a potential therapeutic for MASLD and MASH. LOLA functions as a metabolic modulator, targeting ammonia detoxification pathways in the liver and skeletal muscle and facilitating ammonia detoxification via enhanced urea synthesis and glutamine production, while its metabolites, such as glutathione (GSH) and nitric oxide (NO), exhibit antioxidant properties and improve hepatic microcirculation [201]. Preclinical studies conducted in animal models highlighted LOLA’s ability to reduce oxidative stress, lipid peroxidation, and inflammation while improving muscle function and metabolic stability [202,203,204,205]. Clinical trials have shown significant reductions in liver enzymes (ALT, AST) and triglycerides and improvements in liver/spleen CT ratios and hepatic microcirculation following LOLA treatment [206,207,208]. Despite these promising findings, limitations in existing studies, such as reliance on serum transaminases as outcome markers, necessitate more extensive, well-controlled trials with robust biomarkers to confirm LOLA’s efficacy and long-term benefits in NAFLD/NASH management.

### 3.3. Enhancing Insulin Sensitivity and Glucose Homeostasis

#### 3.3.1. GLP-1 Receptor Agonists

Improving insulin sensitivity and glucose homeostasis are essential for managing MASH and MASLD, and glucagon-like peptide-1 receptor agonists (GLP-1 RAs) play a critical role in this process [224]. These agents enhance insulin sensitivity by increasing insulin secretion from pancreatic beta cells through the activation of GLP-1 receptors and inhibiting glucagon release from alpha cells, leading to reduced hepatic glucose production and balanced blood glucose levels [321]. At the cellular level, GLP-1 RAs promote glucose uptake in muscle and adipose tissues by increasing the translocation of the GLUT2 and GLUT4 transporter to the cell membrane [322,323]. This is facilitated by a cascade of signaling events initiated by insulin receptor activation, resulting in the increased phosphorylation of insulin receptor substrates (IRS-1/2) and activation of phosphoinositide 3-kinase (PI3K) and protein kinase B (AKT). AKT activation is vital for the movement of GLUT4 vesicles to the cell surface, allowing glucose entry into cells [322,324]. Additionally, GLP-1 RAs enhance downstream insulin signaling pathways involving phosphoinositide-dependent kinase-1 (PDK-1) and protein kinase C-zeta (PKC-ζ), which maintain the structural integrity of insulin signaling complexes [324]. GLP-1 RAs mimic the action of the incretin hormone GLP-1, enhancing glucose-dependent insulin secretion, inhibiting glucagon secretion, slowing gastric emptying, and reducing appetite, collectively leading to improved glycemic control and weight loss [224].

GLP-1 RAs, including liraglutide, semaglutide, dulaglutide, and exenatide, enhance insulin sensitivity through various mechanisms. Liraglutide stimulates insulin secretion from pancreatic beta cells and inhibits glucagon release from alpha cells, reducing hepatic glucose production and balancing blood glucose levels [209]. It also induces weight loss by reducing appetite and food intake via GLP-1 receptor activation in the hypothalamus [210,211]. Liraglutide targets pathways such as the GLP-1 receptor pathway, insulin signaling (IRS-1/2, PI3K, and AKT), AMPK, and NF-κB, resulting in enhanced fatty acid oxidation, reduced lipogenesis, and decreased hepatic inflammation [325]. Pre-clinical studies using obese and diabetic mice (e.g., db/db mice and high-fat-diet-induced obese mice), Zucker diabetic fatty rats, HepG2 cells, and primary hepatocytes from human and rodent livers have demonstrated liraglutide’s efficacy in reducing hepatic steatosis and improving insulin sensitivity and liver health markers [211,212,213,214,215].

Semaglutide similarly improves insulin sensitivity by increasing insulin secretion and inhibiting glucagon release, promoting weight loss through central mechanisms in the brain [216]. It reduces hepatic fat content by enhancing insulin sensitivity and promoting fatty acid oxidation [217]. Semaglutide targets the GLP-1 receptor, insulin signaling, AMPK, TGF-β, and inflammatory pathways [218,219]. Phenotypic effects include weight loss, reduced liver fat, improved glycemic control, and decreased liver fibrosis. Pre-clinical studies in high-fat-diet-induced obese mice, diabetic mouse models (e.g., db/db mice), and primary hepatocytes have shown semaglutide’s ability to reduce liver fat and inflammation, improve hepatic insulin sensitivity, and slow fibrosis progression [217,220].

Dulaglutide enhances insulin sensitivity by stimulating insulin secretion and suppressing glucagon release, improving glycemic control and reducing hepatic glucose production [221]. It promotes weight loss by decreasing appetite and increasing satiety through GLP-1 receptor activation in the CNS [222]. Dulaglutide targets the GLP-1 receptor, insulin signaling, AMPK, and NF-κB pathways, reducing hepatic inflammation and promoting fatty acid oxidation [223]. Phenotypic effects include reduced liver steatosis, improved liver enzyme levels, weight loss, and enhanced glycemic control. Pre-clinical models using primary hepatocytes from human and rodent livers and rodent models, including high-fat-diet-induced obese mice and diabetic mouse models (e.g., db/db mice), have demonstrated dulaglutide’s efficacy in reducing hepatic steatosis and improving liver enzyme levels and insulin sensitivity [326,327,328].

Exenatide increases insulin sensitivity by enhancing insulin secretion and inhibiting glucagon release, aiding in weight loss through central GLP-1 receptor activation [224]. It improves liver enzyme levels and reduces liver fat content by promoting insulin sensitivity and fatty acid oxidation [146]. Exenatide targets the GLP-1 receptor, insulin signaling, AMPK, and inflammatory pathways, reducing hepatic inflammation and oxidative stress [225]. Phenotypic effects include improved liver enzyme levels, reduced liver fat, weight loss, and better glycemic control [224]. Pre-clinical studies in HepG2 cells, primary hepatocytes from human and rodent livers, high-fat-diet-induced obese mice, and diabetic rat models (e.g., ZDF rats) have shown exenatide’s potential to improve liver health by reducing hepatic steatosis and inflammation [226,227]. Clinical trials, such as the EXSCEL study (NCT01144338), have investigated exenatide’s long-term effects on cardiovascular outcomes, glycemic control, and liver health in T2DM patients, with additional studies exploring its benefits in NAFLD patients [228,229].

#### 3.3.2. Sodium–Glucose Cotransporter 2 Inhibitors

Sodium–glucose cotransporter 2 (SGLT2) inhibitors are a class of medications that have become essential in managing hyperglycemia, especially in patients with T2DM [242]. Unlike GLP-1 receptor agonists, which enhance insulin secretion and sensitivity, SGLT2 inhibitors complement diabetes management by reducing glucose reabsorption in the kidneys, promoting glycosuria, and subsequently lowering blood glucose levels. The associated benefits of weight loss and improved insulin sensitivity also contribute to the reduction in liver fat and inflammation [242].

SGLT2 inhibitors function by targeting SGLT2 proteins located in the proximal tubules of the kidneys. These proteins are responsible for reabsorbing approximately 90% of the glucose filtered by the kidneys back into the bloodstream [329]. By inhibiting SGLT2, these medications prevent glucose reabsorption, leading to its excretion in the urine, a process known as glycosuria [329]. This mechanism effectively reduces blood glucose levels. Additionally, the inhibition of SGLT2 results in mild osmotic diuresis, contributing to weight loss and lowering blood pressure, thus providing cardiovascular benefits [329]. Furthermore, SGLT2 inhibitors positively influence metabolic pathways related to insulin sensitivity and lipid metabolism, offering a multifaceted approach to diabetes management by controlling blood glucose levels and providing ancillary benefits such as weight reduction and cardiovascular protection [242].

Empagliflozin, a prominent SGLT2 inhibitor, functions by inhibiting SGLT2 proteins in the proximal tubules of the kidneys, thereby reducing glucose reabsorption and promoting glycosuria [230]. This results in lower blood glucose levels and additional metabolic benefits [230]. Empagliflozin specifically targets the insulin signaling pathway by reducing the formation of lipotoxic intermediates such as diacylglycerols (DAG) and increasing the expression of nuclear factor erythroid 2-related factor 2 (Nrf2) and fibroblast growth factor 21 (FGF21), both of which are involved in lipid metabolism and improving insulin sensitivity [231]. The primary phenotypic targets of empagliflozin include reducing liver steatosis and fibrosis, improving insulin sensitivity, and decreasing hepatic triacylglycerol levels. Pre-clinical studies have demonstrated that empagliflozin reduces hepatic fat accumulation and improved insulin resistance in various rodent models of MASLD, including high-fat-diet-induced obese mice and humans [232,233].

Canagliflozin, another SGLT2 inhibitor, works by inhibiting SGLT2 proteins, thus reducing glucose reabsorption in the kidneys and promoting glycosuria [234]. This mechanism lowers blood glucose levels [235]. Canagliflozin targets pathways involved in de novo lipogenesis and fatty acid oxidation by reducing the expression of sterol regulatory element-binding proteins (SREBP1) and influencing the mammalian target of the rapamycin (mTOR) signaling pathway [236]. It aims to reduce hepatic fat content and inflammation while improving insulin sensitivity [236]. Pre-clinical studies in rodent models, such as high-fat-diet-induced obese mice, have demonstrated canagliflozin’s efficacy in reducing hepatic lipid content and inflammation, confirming its potential role in managing MASLD by modulating key metabolic pathways [235,236,237].

Dapagliflozin reduces glucose reabsorption in the kidneys by inhibiting SGLT2 proteins, promoting glycosuria, and lowering blood glucose levels [238]. It targets the insulin signaling pathway, the AMPK pathway, and TGF-β signaling pathway, all of which are involved in fatty acid oxidation, inflammation reduction, and fibrosis mitigation [239]. Dapagliflozin aims to reduce hepatic fat, inflammation, and fibrosis while improving insulin sensitivity and glycemic control. Pre-clinical studies in rodent models, including high-fat-diet-induced obese mice and diabetic mouse models (e.g., db/db mice), have demonstrated dapagliflozin’s ability to reduce hepatic steatosis, inflammation, and liver fat content, highlighting its potential in managing MASLD [239,240,241].

Ipragliflozin works by inhibiting SGLT2 proteins in the kidneys, reducing glucose reabsorption, and promoting glycosuria, thereby lowering blood glucose levels [243]. It targets pathways involved in fatty acid oxidation and inflammation by modulating the AMPK pathway and reducing nuclear factor kappa-light-chain-enhancer of activated B cells (NF-κB) signaling, which are crucial for reducing hepatic inflammation and oxidative stress [244]. Ipragliflozin reduces hepatic steatosis, hyperlipidemia, oxidative stress, and improves insulin sensitivity [242]. Pre-clinical studies have shown significant effects of ipragliflozin in reducing hepatic steatosis and inflammation in animal models of MASLD and patients with type 2 diabetes (NCT02564211, NCT02650609), demonstrating its efficacy in promoting fatty acid oxidation and reducing hepatic lipid accumulation and oxidative stress [245,246]. SGLT2 inhibitors offer a comprehensive approach to diabetes management, extending beyond glucose control to include significant metabolic benefits such as weight reduction, cardiovascular protection, and improvements in liver health. Each SGLT2 inhibitor—empagliflozin, canagliflozin, dapagliflozin, and ipragliflozin—targets specific pathways and mechanisms, providing unique benefits in the management of T2DM and MASLD.

### 3.4. Targeting NASH Microbiome

#### 3.4.1. Probiotics

Several therapeutic strategies targeting the gut microbiome are being explored for treating MASLD, with promising potential to modulate disease progression [23,330,331,332,333]. Probiotics, such as Lactobacillus and Bifidobacterium species, have shown beneficial effects in clinical studies, including reductions in liver fat content and improvements in liver enzyme levels [247,248]. Synbiotics, which combine probiotics with prebiotics like inulin and fructo-oligosaccharides (FOS), offer an enhanced approach by providing a supportive environment for beneficial bacteria, thereby improving gut microbiota composition and reducing hepatic fat accumulation [249]. Fecal Microbiota Transplantation (FMT) is another innovative approach, involving the transfer of healthy donor fecal matter to restore a balanced gut microbiome; early trials have demonstrated improvements in insulin sensitivity and liver fat reduction [252,253].

#### 3.4.2. Dietary Intervention

Dietary interventions, notably the Mediterranean diet, which is rich in fiber, polyphenols, and healthy fats, have also been linked to better liver health outcomes [333]. Additionally, postbiotics, such as short-chain fatty acids (SCFAs) produced during the bacterial fermentation of dietary fibers, exhibit anti-inflammatory properties and enhance gut barrier function [250]. While antibiotics like rifaximin have been used to decrease endotoxin-producing bacteria and improve liver function, their long-term use remains controversial due to potential resistance issues [251]. Collectively, these microbiome-targeting strategies highlight a multifaceted approach to managing MASLD, emphasizing the importance of a healthy gut microbiota in liver disease treatment.

## 4. Conclusions

In 2020, MASLD was re-defined to compass the importance of metabolic dysfunction which is interconnected with liver disease. Many believe that changing the terminology and gaining better knowledge of the pathogenesis of MASLD/MASH will advance drug discovery in new therapeutic areas for the treatment of this complex disease. While the recent FDA approval of resmetirom is a major advancement for the treatment of MASLD and MASH, its modest response rate indicates room for improvement; opening opportunities for other drug candidates or combination therapies with synergistic outcomes or targeting different stages of MASH could be effective treatment strategies. Despite this progress in therapeutic development, challenges remain in diagnosing and treating these conditions where public awareness is low. Hence, the early diagnosis of the disease and intervention are crucial for better patient outcomes. Future research and development in non-invasive diagnostic tools or blood-based biomarkers, which are promising alternatives to liver biopsies, may potentially improve diagnostic accuracy, and the discovery of predictive biomarkers could also enhance personalized treatment approaches.

Overall, while resmetirom’s approval is a significant milestone for the treatment of MASLD/MASH, ongoing research and development are essential to address the remaining challenges in managing MASLD/MASH. The multifactorial nature of MASH and MASLD necessitates a poly-pharmacological approach to ameliorate the hepatic and systemic manifestations of these conditions effectively. By targeting lipid metabolism, oxidative stress, inflammation, and insulin sensitivity, along with dietary interventions, these therapeutic mechanisms provide a comprehensive strategy for managing and potentially reversing the progression of MASH and MASLD.

## Figures and Tables

**Table 1:** Class of considered drugs for treating MASH/MASLD.

Category	Target	Drug Name	Target/Mechanism	Features/Benefits
Targeting Lipid Metabolism and Reducing Lipotoxicity	ACC1/2 inhibitor	PF-05221304 [105,106,107]	Inhibits acetyl-CoA carboxylase (ACC), targeting lipid synthesis pathways	Small molecule; dose-dependent declines in liver fat; minimal adverse events; phase 2a trials showed effectiveness in reducing liver fat percentage
		IMA-1 [108]	Inhibits indoleamine 2,3-dioxygenase 1 (IDO1), modulating tryptophan metabolism and immune response	Small molecule; does not induce hyperlipidemia; halting diet induced NASH progression in male mice and macaque models; avoids side effects
	DGAT2 inhibitor	PF-06865571 [105,106]	Inhibits diacylglycerol O-acyltransferase 2 (DGAT2), targeting triglyceride synthesis	Small molecule; combined therapy with PF-05221304 showed significant reduction in liver fat
	FGF analogs	Aldafermin (NGM282) [109]	Analog of FGF19, modulating bile acid synthesis, glucose metabolism, and lipid metabolism	Peptide; phase 2 study showed fibrosis improvement without worsening NASH
		Efruxifermin (EFX, also known as AKR-001 or AMG876) [110]	Fc-FGF21 fusion protein, targeting FGF21 receptor pathways involved in glucose and lipid metabolism	Peptide; improved pharmacokinetics and pharmacodynamics; showed potential in improving glycemic control; has good safety profile; demonstrated antifibrotic effects meeting FDA criteria for phase 3 trials
		BFKB8488A [111]	Monoclonal antibody targeting FGFR1c/KLB, involved in metabolic regulation	Peptide; effective in reducing liver fat and enhancing metabolic parameters; favorable safety profile
	FXR agonists	Obeticholic Acid (OCA) [112,113,114,115,116,117,118,119]	Semi-synthetic bile acid, potent FXR agonist	Regulates bile acid synthesis, improves insulin sensitivity; reduces liver fat, inflammation, and fibrosis.; demonstrated significant histological improvements in clinical trials but was associated with side effects like pruritus and altered lipid profiles; investigated in phase 2 and 3 trials
		Tropifexor (LJN452) [120,121,122,123,124]	Non-bile acid FXR agonist	Potent FXR agonist with significant reductions in hepatic triglycerides, liver fibrosis, and inflammation in preclinical studies; phase 2 trials showed efficacy in reducing liver enzymes and liver fat but with side effects like pruritus and changes in lipid profiles
		Cilofexor (GS-9674) [125,126,127,128,129]	Non-bile acid FXR agonist	Shown to reduce hepatic steatosis and improve liver biochemistry in preclinical and phase 2 studies; demonstrated potential to reduce fibrosis and portal hypertension; side effects include pruritus at higher doses
		Vonafexor (EYP001) [130,131,132]	Non-bile acid FXR agonist	Selective FXR agonist with promising results in preclinical studies, showing reductions in liver fat, fibrosis, and inflammation; phase 2 studies demonstrated reduced liver fat content and liver enzymes; mild pruritus observed at higher doses
	THRβ agonist	Resmetirom [133,134,135,136,137,138,139,140,141]	Agonist of thyroid hormone receptor beta (THR-β), influencing cholesterol and lipid metabolism	Small molecule; enhances fatty acid oxidation and reduces lipogenesis in liver; promotes mitochondrial biogenesis and mitophagy and reduces lipid accumulation, inflammation, and fibrosis; decreases triglyceride synthesis, promotes LDL cholesterol uptake, and enhances bile acid synthesis, leading to improved lipid profiles by upregulating deiodinase type 1 (DIO1); improves intrahepatic thyroid hormone signaling and lipid metabolism, reducing hepatic lipotoxicity; first-ever FDA-approved treatment for NASH
	PPAR agonists	Lanifibranor [142,143,144]	Pan-PPAR agonist (PPARα, PPARδ, and PPARγ), targeting lipid and glucose metabolism	Small molecule; clinical studies in highly active NASH showed significant liver histology improvements without worsening fibrosis
		Elafibranor [145,146]	Dual PPARα/δ agonist, affecting lipid metabolism, inflammation, and glucose homeostasis	Small molecule; showed improving NASH-related histological parameters, failed to meet primary endpoint of NASH resolution without fibrosis progression, leading to its discontinuation
		Saroglitazar [147,148]	Dual PPARα/γ agonist, modulating lipid and glucose metabolism	Small molecule; improved liver enzyme levels, hepatic fat content, and metabolic parameters; approved in India for NASH, with pending broader international approval
		Pemafibrate [149,150,151]	Selective PPARα modulator, targeting lipid metabolism	Small molecule; showed benefits in hepatic inflammation, fibrosis, and biochemical scores
	SCD1 modulators	Dexmedetomidine (DEX) [152]	Selective α2-adrenergic agonist, affecting central nervous system and sedation pathways	Small molecule; improved insulin sensitivity and reduced hepatic steatosis and inflammation in NAFLD mice
	Antisense oligonucleotides (ASOs)	AZD2693 (ION839) [153,154]	Targets PNPLA3 gene, specifically I148M variant linked to increased liver triglyceride accumulation	Reduces expression of mutated PNPLA3 protein, lowering hepatic lipid storage; shown to reduce liver triglyceride levels and alleviate steatosis in animal models; targeted NASH patients with PNPLA3 I148M variant, which is genetic risk factor in phase 2 trials
		DGAT2 ASO (ION224) [155,156,157,158,159,160]	Targets DGAT2 enzyme, involved in final step of triglyceride synthesis	Reduces hepatic lipid accumulation by inhibiting triglyceride synthesis; shown to improve liver histology in preclinical studies, reducing inflammation and fibrosis; phase 2 trials in NASH patients showed improvements in liver histology without worsening fibrosis
		STK25 ASO [161,162,163]	Targets STK25, regulator of lipid metabolism and insulin sensitivity	Reduces liver fat accumulation and improves β-oxidation by decreasing STK25 activity; preclinical studies demonstrated significant reductions in liver steatosis and improved insulin sensitivity; currently in preclinical development
		HSD17B13 ASO (ARO-HSD) [164,165,166,167]	Targets HSD17B13 gene, leveraging protective effects of loss-of-function variants associated with reduced risk of liver diseases	Reduces inflammation and fibrosis by silencing HSD17B13; early clinical trials showed reduced hepatic HSD17B13 expression and protein levels, with promising results in mitigating liver damage in MASLD and MASH patients
Reducing Oxidative Stress and Inflammation	ASK1 inhibitor	Selonsertib [168,169,170]	Inhibits apoptosis signal-regulating kinase 1 (ASK1), involved in inflammation and apoptosis pathways	Small molecule; reduces hepatic macrophage and stellate cell activation, thereby decreasing hepatic inflammation and fibrosis
	CCR antagonists	Cenicriviroc (CVC) [171,172,173]	Dual CCR2/CCR5 antagonist, targeting inflammatory and fibrotic pathways	Small molecule; reduces influx and activation of these immune cells, thereby mitigating hepatic inflammation
	Galectin-3 inhibitor	Belapectin [174,175]	Inhibits galectin-3, targeting fibrosis and inflammation pathways	Large molecule galactoarabino-rhamnogalacturonan polysaccharide inhibitor derived from natural sources; prevents formation of oligomeric structures and lattice-like assemblies that are essential for activating pro-fibrotic signaling pathways, thereby halting fibrosis and reducing liver inflammation
		GB1107 [176,177]	Inhibits galectin-3, targeting fibrosis and inflammation pathways	Small molecule thiogalactoside inhibitor targeting carbohydrate recognition domain (CRD) of galectin-3
		GB1211 [178]	Inhibits galectin-3, targeting fibrosis and inflammation pathways	Small molecule; analog of GB1107
	RXFP1 agonists	Relaxin and its mimetics [179,180,181,182,183,184,185,186,187,188,189,190,191,192,193,194,195,196,197,198,199,200]	Activates RXFP1	Induces multiple effects including anti-fibrosis, anti-inflammatory, vasoprotective actions in various organs
	L-ornithine L-aspartate (LOLA) [201,202,203,204,205,206,207,208]		Metabolic modulator, targeting ammonia detoxification pathways in liver and skeletal muscle	Small molecule; reduces hyperammonemia, enhances urea cycle function; lowers oxidative stress; improves microcirculation
Enhancing Insulin Sensitivity and Glucose Homeostasis	GLP-1 receptor agonist	Liraglutide [209,210,211,212,213,214,215]	GLP-1 receptor agonist, enhancing insulin secretion and glucose homeostasis	Peptide; promotes glucose uptake in muscle and adipose tissues; enhances glucose-dependent insulin secretion and inhibits glucagon secretion, slowing gastric emptying, and reducing appetite, collectively leading to improved glycemic control and weight loss
		Semaglutide [216,217,218,219,220]	GLP-1 receptor agonist, enhancing insulin secretion and glucose homeostasis	Peptide; reduces liver fat and inflammation, improves hepatic insulin sensitivity, and slows fibrosis progression through central mechanisms in brain
		Dulaglutide [221,222,223]	GLP-1 receptor agonist fused to Fc fragment, enhancing insulin secretion and glucose homeostasis	Peptide; enhances insulin sensitivity by stimulating insulin secretion and suppressing glucagon release; improves glycemic control and reducing hepatic glucose production; promotes weight loss by decreasing appetite
		Exenatide [146,224,225,226,227,228,229]	Synthetic version of exendin-4, GLP-1 receptor agonist, enhancing insulin secretion and glucose homeostasis	Peptide; targets GLP-1 receptor, insulin signaling, AMPK, and inflammatory pathways, reducing hepatic inflammation and oxidative stress
	SGLT2 inhibitor	Empagliflozin [230,231,232,233]	SGLT2 inhibitor specifically targets insulin signaling pathway	Small molecule; pre-clinical studies demonstrated reduction in hepatic fat accumulation, improved insulin resistance in various rodent models of NAFLD, including high-fat-diet-induced obese mice and humans
		Canagliflozin [234,235,236,237]	SGLT2 inhibitor targets pathways involved in de novo lipogenesis and fatty acid oxidation	Small molecule; reduced hepatic lipid content and inflammation by modulating key metabolic pathways in pre-clinical rodent models
		Dapagliflozin [238,239,240,241]	SGLT2 inhibitor targets insulin signaling pathway involved in fatty acid oxidation, inflammation reduction, and fibrosis mitigation	Small molecule; reduce hepatic steatosis, inflammation, and liver fat content in high-fat-induced obese and diabetic mice models
		Ipragliflozin [242,243,244,245,246]	SGLT2 inhibitor targeting AMPK pathway and reducing nuclear factor kappa-light-chain-enhancer of activated B cells (NF-κB) signaling for reducing hepatic inflammation and oxidative stress	Small molecule; significantly reduced hepatic steatosis and inflammation in animal models of NAFLD and patients with type 2 diabetes in pre-clinical studies
Targeting NASH Microbiome	Probiotics	Lactobacillus [247] and Bifidobacterium species [248]; probiotics and prebiotics such as inulin and FOS [249]	Modulates gut microbiota to improve liver health	Reduce liver fat content and improve liver enzyme levels; shown to improve liver health outcomes in clinical studies
	Postbiotics	Short-chain fatty acids (SCFAs) [250]	Anti-inflammatory compounds produced during bacterial fermentation of dietary fibers	Enhance gut barrier function and exhibit anti-inflammatory properties, contributing to better liver health
	Dietary intervention	Mediterranean diet, rich in fiber, polyphenols, and healthy fats	Promotes liver health through gut-friendly dietary patterns	Improves liver function by promoting healthy gut microbiota; linked to reduced liver fat and improved liver health outcomes in patients with NASH
	Antibiotics	Rifaximin [251]	Reduces endotoxin-producing bacteria in gut	Improves liver function by decreasing harmful bacteria; long-term use is controversial due to potential antibiotic resistance
	Fecal Microbiota Transplantation (FMT) [252,253]		Restores balanced gut microbiome through transfer of fecal matter from healthy donors	Improves insulin sensitivity and reduces liver fat, with early clinical trials showing positive outcomes in NASH patients.

CCR = C-C chemokine receptor; FGF = fibroblast growth factor; FOS = fructo-oligosaccharides; FXR = farnesoid X receptor; GLP-1 = glucagon-like peptide-1; PPAR = peroxisome proliferator-activated receptor; SCD1 = stearoyl-CoA desaturase 1; SGLT2 = sodium–glucose cotransporter 2; RXFP1 = relaxin/insulin-like family peptide receptor 1.

## Abbreviations

ACC	Acetyl-CoA carboxylase
ALT	Alanine aminotransferase
AMPK	AMP-activated protein kinase
APOC3	Apolipoprotein C3
ASK1	Apoptosis signal-regulating kinase 1
AST	Aspartate aminotransferase
ASOs	Antisense oligonucleotides
CCR	C-C chemokine receptor
CDCA	Chenodeoxycholic acid
CD98hc	CD98 heavy chain
CRD	Carbohydrate recognition domain
DGAT2	Diacylglycerol acyltransferase 2
DEX	Dexmedetomidine
DNL	De novo lipogenesis
ER	Endoplasmic reticulum
FATP5	Fatty acid transport protein 5
FGF4	Fibroblast growth factor 4
FGF19	Fibroblast growth factor 19
FMT	Fecal Microbiota Transplantation
FOS	Fructo-oligosaccharides
FXR	Farnesoid X receptor
G6Pase	Glucose-6-phophatase
GLP-1	Glucagon-like peptide-1
GSH	Glutathione
HDL	High-density lipoprotein
HFD	High-fat diet
HSCs	Hepatic stellate cells
HSD17B13	Hydroxysteroid 17 beta dehydrogenase 13
IDO1	Indoleamine 2,3-dioxygenase 1
IL-6	Interleukin 6
LOLA	L-ornithine L-aspartate
IR	Insulin resistance
IRGM	Immunity-related GTPase family M
LPS	Lipopolysaccharides
MASLD	Metabolic dysfunction-associated steatotic liver disease
MASH	Metabolic dysfunction-associated steatohepatitis
MBOAT7	Membrane-bound O-acyltransferase domain-containing 7
NAFLD	Non-alcoholic fatty liver disease
NAS	NAFLD activity score
NF-κB	Nuclear factor kappa-light-chain-enhancer of activated B
NLRP3	NOD-, LRR-, and pyrin domain-containing protein 3
NO	Nitric oxide
OCA	Obeticholic acid
PEPCK	Phosphoenolpyruvate carbpxykinase
PGC1a	Peroxisome proliferator-activated receptor gamma coactivator 1 alpha
PNPLA3	Patatin-like phospholipase domain-containing protein 3
PPARs	Peroxisome proliferator-activated receptors
PRRs	Pattern recognition receptors
ROS	Reactive oxygen species
RXFP1	Relaxin/insulin-like family peptide receptor 1
SCD1	Stearoyl-CoA desaturase 1
SGLT2	Sodium-glucose cotransporter 2
SOD2	Superoxide dismutase 2
SHP	Small heterodimer partner
SREBP-1c	Sterol regulatory element-binding protein-1c
T2DM	Type 2 diabetes mellitus
TGF-β1	Transforming growth factor beta 1
THR-β	Thyroid hormone receptor beta
TNF-α	Tumor necrosis factor alpha
TM6SF2	Transmembrane 6 superfamily member 2
TMAO	Trimethylamine N-oxide
UPR	Unfolded protein response
VLDL	Very-low-density lipoprotein
